# Chronic post-COVID neuropsychiatric symptoms persisting more than 1 year after infection during the ‘Omicron wave’

**DOI:** 10.1192/bjo.2025.10078

**Published:** 2025-07-25

**Authors:** Steven Wai Ho Chau, Timothy Mitchell Chue, Tsz Ching Lam, Yee Lok Lai, Rachel Ngan Yin Chan, Paul W. C. Wong, Shirley Xin Li, Yaping Liu, Joey Wing Yan Chan, Paul Kay-sheung Chan, Christopher Koon-Chi Lai, Thomas W. H. Leung, Yun Kwok Wing

**Affiliations:** Department of Psychiatry, Faculty of Medicine, The Chinese University of Hong Kong, Hong Kong SAR; Li Chiu Kong Family Sleep Assessment Unit, Department of Psychiatry, Faculty of Medicine, The Chinese University of Hong Kong, Hong Kong SAR; Department of Social Work and Social Administration, Faculty of Social Sciences, The University of Hong Kong, Hong Kong SAR; Department of Psychology, Faculty of Social Science, The University of Hong Kong, Hong Kong SAR; Center for Sleep and Circadian Medicine, The Affiliated Brain Hospital, Guangzhou Medical University, Guangzhou, Guangdong, China; Department of Microbiology, Faculty of Medicine, The Chinese University of Hong Kong, Hong Kong SAR; S.H. Ho Research Centre for Infectious Diseases, Faculty of Medicine, The Chinese University of Hong Kong, Hong Kong SAR; Division of Neurology, Department of Medicine and Therapeutics, Faculty of Medicine, The Chinese University of Hong Kong, Hong Kong SAR; Li Ka Shing Institute of Health Sciences, Faculty of Medicine, The Chinese University of Hong Kong, Shatin, Hong Kong SAR

**Keywords:** Infection, neuropsychiatry, long-COVID, network analysis

## Abstract

**Background:**

The heterogeneity of chronic post-COVID neuropsychiatric symptoms (PCNPS), especially after infection by the Omicron strain, has not been adequately explored.

**Aims:**

To explore the clustering pattern of chronic PCNPS in a cohort of patients having their first COVID infection during the ‘Omicron wave’ and discover phenotypes of patients based on their symptoms’ patterns using a pre-registered protocol.

**Method:**

We assessed 1205 eligible subjects in Hong Kong using app-based questionnaires and cognitive tasks.

**Results:**

Partial network analysis of chronic PCNPS in this cohort produced two major symptom clusters (cognitive complaint–fatigue and anxiety–depression) and a minor headache–dizziness cluster, like our pre-Omicron cohort. Participants with high numbers of symptoms could be further grouped into two distinct phenotypes: a cognitive complaint–fatigue predominant phenotype and another with symptoms across multiple clusters. Multiple logistic regression showed that both phenotypes were predicted by the level of pre-infection deprivation (adjusted *P*-values of 0.025 and 0.0054, respectively). The severity of acute COVID (adjusted *P* = 0.023) and the number of pre-existing medical conditions predicted only the cognitive complaint–fatigue predominant phenotype (adjusted *P* = 0.003), and past suicidal ideas predicted only the symptoms across multiple clusters phenotype (adjusted *P* < 0.001). Pre-infection vaccination status did not predict either phenotype.

**Conclusions:**

Our findings suggest that we should pursue a phenotype-driven approach with holistic biopsychosocial perspectives in disentangling the heterogeneity under the umbrella of chronic PCNPS. Management of patients complaining of chronic PCNPS should be stratified according to their phenotypes. Clinicians should recognise that depression and anxiety cannot explain all chronic post-COVID cognitive symptoms.

The COVID-19 pandemic has had a lasting impact on people’s lives worldwide. Despite the world having been keen to move on from the shadow of the pandemic, there are still many unsolved questions about the long-term consequences of COVID in some patients. Neuropsychiatric symptoms such as fatigue, cognitive impairments, anxiety and depression are among the most common post-COVID symptoms.^1^ These symptoms can be unremitting, lasting for more than 2 years after infection.^2^ An elevated incidence of psychiatric disorders such as depression and anxiety disorder has been found among those who have recovered from COVID-19,^3^ which can cause an increase in the burden on the mental health system. However, it has been challenging to obtain a full understanding of chronic post-COVID neuropsychiatric symptoms (PCNPS) owing to various factors, including variants of the SARS-CoV-2 strain, vaccination status and the use of antiviral drugs. Early studies primarily focused on those infected by pre-Omicron strains and patients admitted to hospital, rather than mild acute COVID cases. Although some studies have suggested that the prevalence of post-COVID symptoms among patients infected by the Omicron variant was lower than that for some of the earlier variants, other studies have disagreed. Moreover, there remain significant knowledge gaps; for example, the heterogeneity of chronic PCNPS in patients infected by Omicron and post-Omicron variants has not been adequately explored. Although PCNPS are often grouped into a single entity, our previous study suggested that chronic PCNPS among those infected by pre-Omicron strains could be clustered into an anxiety–depression cluster and a cognitive complaint–fatigue (CCF) cluster.^4^ The identification of scientifically meaningful symptom clusters and phenotypes among patients will provide a basis for further investigation into the epidemiology and pathophysiology of the phenomenon, as well as guiding treatment. It would be of particular interest to investigate the heterogeneity of chronic PCNPS among patients infected with Omicron and post-Omicron strains to determine whether it follows a similar pattern to that observed in pre-Omicron patients. In addition, few studies of the risk factors for chronic PCNPS have considered biological factors together with socioeconomic factors, and these factors potentially confound each other.

The primary aim of our study was to explore the relationships of chronic PCNPS exhibited by individuals who were infected during the 2022 ‘Omicron wave’ in Hong Kong. The secondary aim of the study was to explore phenotypes among patients suffering from chronic PCNPS and identify their distinct clinical trajectories and risk factors. The key hypotheses of our study were (a) chronic PCNPS in patients infected with SARS-CoV-2 more than 1 year ago during the Omicron wave would show a similar clustering pattern of symptoms to patients infected with pre-Omicron strains (derived from our previous study); and (b) these chronic PCNPS would be associated with (i) clinical risk factors such as the severity of the acute infection, (ii) socioeconomic status, e.g. level of deprivation, and (iii) pre-infection vaccination status.

## Method

The present study is part of the ‘Long-term mental and brain health effects of COVID-19 from the Omicron strains among adult patients’ study. The key hypotheses and outcome measures were pre-registered (https://doi.org/10.1186/ISRCTN11876145). We recruited participants from the community in Hong Kong via online advertisement. The inclusion criteria were: (a) self-report history of a first SARS-CoV-2 infection confirmed by PCR or rapid antigen test that occurred after January 2022 (i.e. after the Omicron variant became dominant in Hong Kong^5^); (b) the infection occurred at least 1 year before the study; and (c) age between 18 and 65 years. Participants who provided consent underwent a panel of assessments using an app created specifically for this study by our team, which included (a) a detailed questionnaire on demographic information and socioeconomic and health status at two time points: December 2021 and recruitment; (b) a COVID symptoms checklist that comprised 16 neuropsychiatric items and 26 non-neuropsychiatric items; (c) standardised measures of mental health, sleep and health-related quality of life; (d) a 7-day sleep diary; and (e) app-based cognitive tasks focusing on key domains (concentration, psychomotor speed and working memory) related to the phenomenon of ‘brain fog’ (see the supplementary material available at https://doi.org/10.1192/bjo.2025.10078).

The authors assert that all procedures contributing to this work comply with the ethical standards of the relevant national and institutional committees on human experimentation and with the Helsinki Declaration of 1975, as revised in 2013. All procedures involving human participants or patients were approved by the Joint CUHK NTEC Clinical Research Ethic Committee (ref. no.: 2022.362) and the Central Hospital Authority Institutional Review Board (ref. no.: CIRB-2022-006-1).

### Data analysis

Similar to our previous work, we built a regularised partial correlation network of self-reported chronic PCNPS to explore their relationships and clustering patterns.^6^ To minimise the instability of the network, we excluded symptoms reported by fewer than 1% of the participants. After network estimation, we used the walktrap algorithm to identify clusters within the symptom network.^7^ We used the community assortativity (R_com_) metric, a bootstrapping procedure, to measure the robustness of community assignment by the walktrap algorithm. Community assignments were deemed to be robust if R_com_ was greater than 0.5.^8^ We used R packages bootnet, IsingFit, igraph, ggraph and plyr to perform the network estimation process, and asnipe and assortnet for assortativity metric estimation. In our previous work, we found that patients with fewer than four chronic PCNPS had similar levels of mental distress to healthy control participants.^4^ In the present study, therefore, we first defined a low-symptom-load group (LSL) using this cut-off. We then used a Bernoulli mixture model (BMM) to explore phenotypes among participants with four or more chronic PCNPS on the basis of their symptom profile. We ran the BMM fitting algorithm for cluster numbers from 1 to 10, with 1000 repetitions for each cluster number, and selected the best-fitting model using the Bayesian information criterion. We used the flexmix R package to fit the BMM.^9^

We tested for univariate between-group differences in demographic characteristics, socioeconomic and health status, and symptom scores among subgroups using non-parametric statistical tests. If the variable was categorical, Pearson’s chi-squared test was used; if there were significant statistical differences among the groups, Pearson’s chi-squared test was also for *post hoc* pairwise comparison tests, but Bonferroni correction was applied to the test results to counteract the multiple comparisons problem. The same method was applied to ordinal and numerical variables, except that the Kruskal–Wallis H-test and Dunn’s test (*post hoc*) were used. We used multinomial logistic regression to predict participants’ membership of phenotypes discovered by the BMM model, with the LSL group as the reference group.

The lead author and manuscript guarantor (S.W.H.C.) affirms that the manuscript is an honest, accurate and transparent account of the study being reported; that no important aspects of the study have been omitted; and that any discrepancies from the study as planned have been explained.

## Results

We recruited 1273 participants, of whom 1205 completed the assessments. The mean age of the subjects was 38.8 years, 54.9% were female, and 2.2% of the sample were of non-Chinese ethnicity. The participants had their first SARS-CoV-2 infection between 2 January 2022 and 10 September 2022, and 24.6% had moderate-to-severe acute COVID (defined as having symptoms of pneumonia and/or requiring oxygen therapy). Before the index infection, 15.0 and 5.4% of participants had had at least one self-reported medical or psychiatric comorbidity, respectively.

### Network analysis

**Fig. 1:**
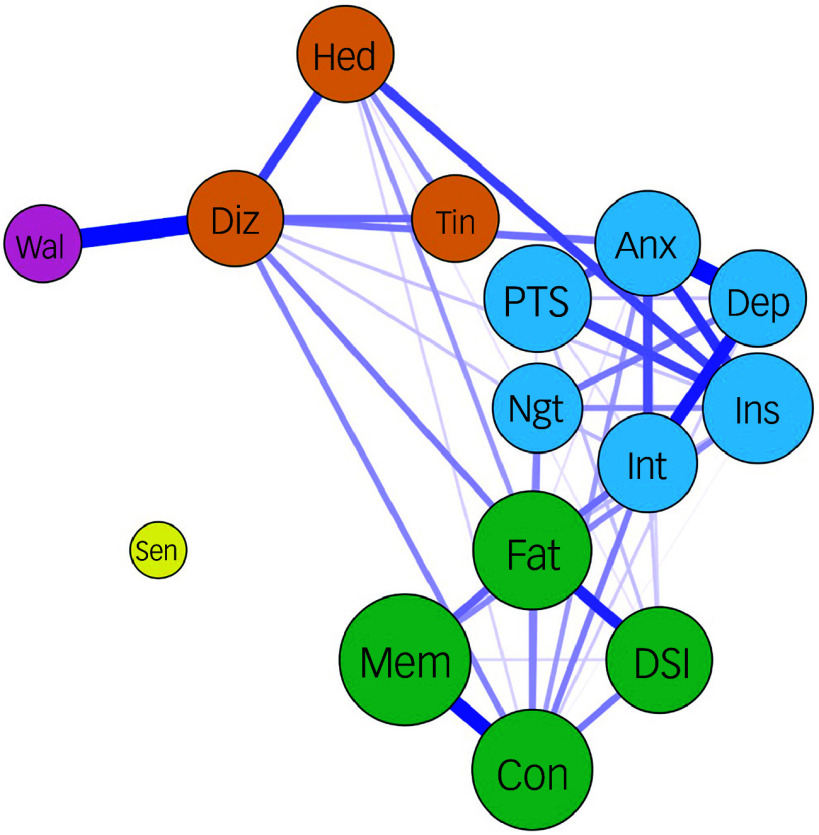
Partial correlation network of chronic post-COVID neuropsychiatric symptoms. Fat, fatigue; Con, inability to concentrate; Mem, memory problems; DSl, daytime sleepiness; Anx, feeling anxious; Dep, feeling depressed; Int, loss of interest or pleasure; PTS, COVID-related post-traumatic stress symptoms; Ins, insomnia; Ngt, frequent nightmare; Hed, headache; Diz, dizziness; Tin, tinnitus; Wal, imbalanced walking; Sen, loss or change to your sense of taste and smell. The colour of the node represents the cluster they belong to. The thickness of the edge represents the strength of the partial correlation between the nodes. The sizes of the circles correlate with the frequency (log scale) of the symptoms they represent.

The most frequently reported chronic PCNPS in our cohort were memory problems (39.2%), inability to concentrate (23.9%), fatigue (21.8%), insomnia (14.7%), post-traumatic stress (12.5%, as measured by the revised version of the Impact of Event Scale), daytime sleepiness (12.1%) and feeling anxious (11.5%) (Table 1), similar to those reported in our pre-Omicron cohort.^4^ Our partial correlation network analysis demonstrated the presence of two major symptom clusters (the CCF cluster and an anxiety–depression cluster) and a minor cluster (headache–dizziness cluster) (Fig. 1). The clustering pattern and cluster membership were very similar to those found in the pre-Omicron cohort in our previous work.^4^

**Table 1:** Top ten most frequently reported chronic post-COVID neuropsychiatric symptoms (*N* = 1205)

Symptom	Frequency	%
Memory problems	472	39.2
Inability to concentrate	288	23.9
Fatigue	263	21.8
Insomnia	177	14.7
Post-traumatic stress	151	12.5
Daytime sleepiness	146	12.1
Feeling anxious	138	11.5
Loss of interest or pleasure	104	8.6
Feeling depressed	93	7.7
Headache	92	7.6

### Phenotyping using BMM model

**Fig. 2:**
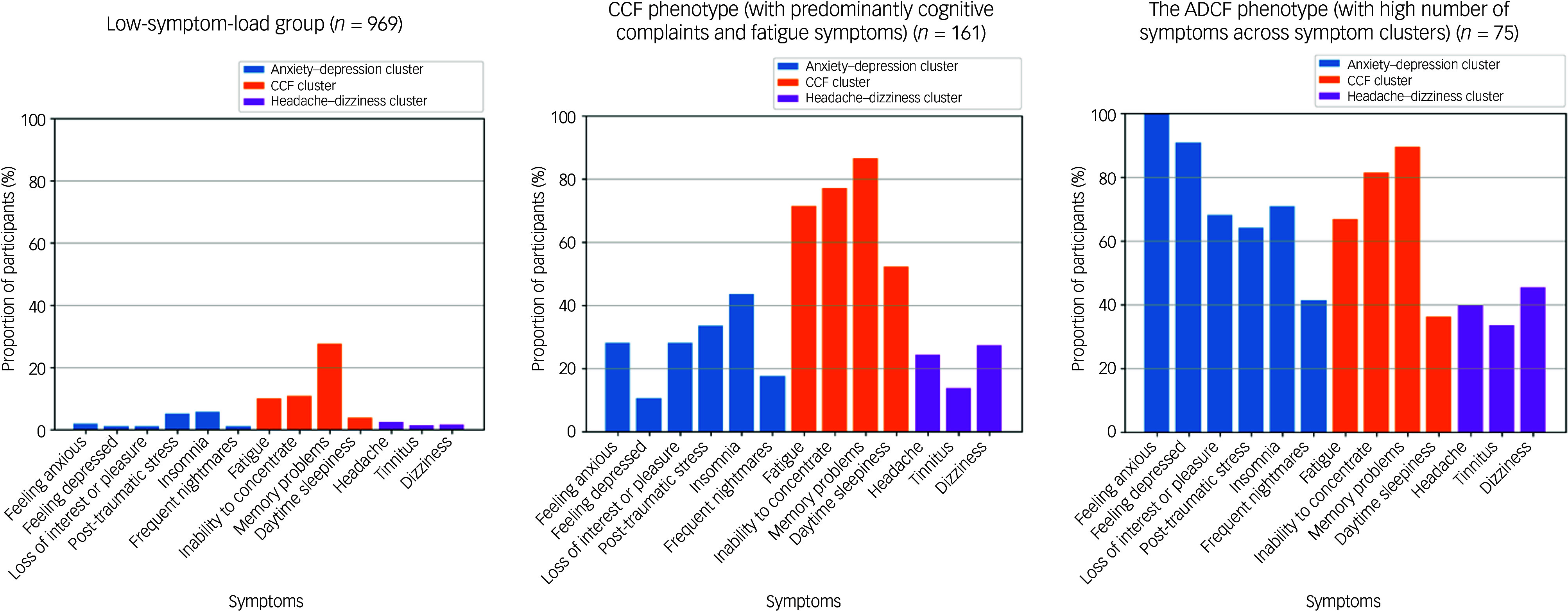
Symptom profiles of the low-symptom-load group, the cognitive complaints–fatigue (CCF) phenotype and the ADCF (high number of symptoms across the anxiety–depressive and CCF clusters) phenotype.

The best BMM model resulted in two phenotypes among those with high symptom load, namely the CCF phenotype (*n* = 161) and another type with a high number of symptoms across the anxiety–depressive and CCF clusters (the ADCF phenotype, *n* = 75) (Fig. 2).

### Univariate comparisons across the LSL, CCF and ADCF groups

The LSL, CCF, and ADCF groups had similar age and gender ratios. The LSL group had the lowest number of medical illnesses before infection (mean 0.2 *v*. 0.4 *v*. 0.3 for LSL *v*. CCF *v*. ADCF, respectively; *P* < 0.001; *post hoc* test: LSL < CCF, LSL < ADCF) and the lowest percentage of psychiatric illnesses (4 *v*. 10 *v*. 11% for LSL *v*. CCF *v*. ADCF, respectively; *P* = 0.0014; *post hoc* test: LSL < CCF, LSL < ADCF), and they were the least deprived as measured by the Deprivation Index^10^ (median 0 *v*. 1 *v*. 2 for LSL *v*. CCF *v*. ADCF, respectively; *P* < 0.001; *post hoc* test LSL < CCF, LSL < ADCF) (Table 2). The LSL group also had the fewest participants who had moderate-to-severe acute COVID (22 *v*. 35 *v*. 37% for LSL *v*. CCF *v*. ADCF, respectively; *P* < 0.001; *post hoc* test LSL < CCF, LSL < ADCF) and the shortest total duration of pandemic-related home quarantine (median 7 *v*. 10 *v*. 11 for LSL *v*. CCF *v*. ADCF, respectively; *P* < 0.001; *post hoc* test LSL < CCF, LSL < ADCF). Moreover, the LSL group had the fewest post-infection emergent physical and mental health problems. By contrast, the CCF and ADCF groups had significantly more post-infection diagnosed psychiatric illnesses (1 *v*. 4 *v*. 8% for LSL *v*. CCF *v*. ADCF, respectively; *P* < 0.001; *post hoc* test LSL < CCF, LSL < ADCF) and new suicidal ideation (3 *v*. 9 *v*. 17% for LSL *v*. CCF *v*. ADCF, respectively; *P* < 0.001; *post hoc* test LSL < CCF, LSL < ADCF) compared with the LSL group, and the CCF group had more newly diagnosed medical problems (mean 0.06 *v*. 0.16 *v*. 0.11 for LSL *v*. CCF *v*. ADCF, respectively; P ≤ 0.001; *post hoc* test LSL < CCF). The ADCF group showed a significant increase in deterioration in socioeconomic status compared with the LSL group, with increases in both deprivation (mean −0.2 *v*. 0.1 *v*. 0.6 for LSL *v*. CCF *v*. ADCF, respectively; *P* < 0.001; *post hoc* test LSL < ADCF) and domestic violence (1 *v*. 2 *v*. 4% for LSL *v*. CCF *v*. ADCF, respectively; *P* = 0.018; *post hoc* test LSL < ADCF) (Table 2).

**Table 2:** Comparisons of demographic factors; pre-infection physical health, mental health and socioeconomic factors; clinical factors related to infection; psychosocial stressors secondary to COVID; post-infection changes in physical health, mental health, sleep health and socioeconomic factors; and current mental well-being, health-related quality of life and app-based cognitive task performance among the low-symptom-load group, CCF phenotype and ADCF phenotype

Variable	Low-symptom-load group	CCF phenotype (with predominantly cognitive complaints–fatigue cluster symptoms)	ADCF phenotype (with high numbers of symptoms across symptom clusters)	*P*-value	*Post hoc*
Demographic factors					
Age in years, mean (s.d.)	38.5 (12.5)	40.1 (11.5)	39.2 (11.4)	0.19	
Female gender, %	53.6	62.1	57.3	0.12	
Non-Chinese ethnicity, %	2.4	3.7	1.3	0.48	
Tertiary education, %	76.9	71.4	64.0	0.021	LSL > ADCF
Pre-infection physical health					
Smoker, %	5.9	8.1	6.7	0.56	
Number of known medical illnesses, mean (s.d.)	0.17 (0.52)	0.42 (0.83)	0.31 (0.57)	<0.001	LSL < CCF LSL < ADCF
Pre-infection mental health					
Any known mental illness, %	4.2	9.9	10.7	0.0014	LSL < CCF LSL < ADCF
Absence of suicidal ideation, %	93.6	86.3	66.7	<0.001	LSL > CCF > ADCF
Risk of alcoholism, %	1.4	0.6	2.7	0.45	
Pre-infection socioeconomic factors					
Deprivation index, median (IQR)	0 (2)	1 (4)	2 (4)	<0.001	LSL < CCF LSL < ADCF
Number of dependent children, median (IQR)	0 (1)	0 (1)	0 (1)	0.22	
Number of elderly dependants, median (IQR)	0 (1)	0 (1)	0 (1)	0.12	
In full-time employment before infection, %	64.3	73.3	68.0	0.076	
Absence of domestic violence, %	96.7	93.2	84.0	<0.001	LSL > ADCF
Clinical factors related to infection					
Moderate-to-severe COVID, %	21.9	34.8	37.3	< 0.001	LSL < CCF LSL < ADCF
Number of doses of mRNA vaccines pre-infection, median (IQR)	2 (2)	2 (2)	2 (3)	0.045	
Number of doses of non-mRNA vaccines pre-infection, median (IQR)	0 (0)	0 (0)	0 (1)	0.22	
Two or more infections, %	22.7	30.4	32.0	0.030	
Any use of antiviral medication, %	5.0	9.9	4.0	0.032	LSL < CCF
Psychosocial stressors secondary to COVID					
Duration of hospital isolation, median (IQR)	0 (0)	0 (0)	0 (0)	0.92	
Duration of quarantine in facilities, median (IQR)	0 (0)	0 (0)	0 (0)	0.64	
Duration of quarantine at home, median (IQR)	7 (7)	10 (7)	11 (7)	<0.001	LSL < CCF LSL < ADCF
Any COVID-related death in close social circle, median (IQR)	0 (0)	0 (0)	0 (0)	0.19	
Post-infection physical health					
Number of new medical diagnoses, mean (s.d.)	0.058 (0.25)	0.16 (0.45)	0.11 (0.31)	<0.001	LSL < CCF
Number of days hospital stay in the past year, mean (s.d.)	0.33 (2.4)	0.57 (3.2)	0.48 (1.4)	0.045	LSL < ADCF
Number of medical consultations in the past year, mean (s.d.)	4.6 (6.9)	8.4 (9.8)	8.8 (12.6)	<0.001	LSL < CCF LSL < ADCF
Body mass index > 30, %	4.9	9.3	4.0	0.058	
Post-infection changes in mental health					
Any newly diagnosed psychiatric illness, %	0.8	3.7	8.0	<0.001	LSL < CCF LSL < ADCF
New suicidal ideation, %	2.6	8.7	17.3	<0.001	LSL < CCF LSL < ADCF
Post-infection sleep health					
Pre-infection perceived sleep demand in hours, median (IQR)	8.0 (1.0)	8.0 (1.0)	8.0 (1.0)	0.72	
Post-infection sleep demand in hours, mean (s.d.)	8.01 (1.45)	8.38 (1.33)	8.55 (2.18)	<0.001	LSL < CCF LSL < ADCF
Actual average sleep time in hours, median (IQR)	6.94 (1.36)	6.33 (1.68)	6.15 (1.58)	<0.001	LSL > CCF LSL > ADCF
Sleep latency in hours, median (IQR)	0.17 (0.17)	0.25 (0.42)	0.33 (0.5)	<0.001	LSL < CCF LSL < ADCF CF < ADCF
Mid-sleep arousal in hours, median (IQR)	0.083 (0.17)	0.13 (0.33)	0.21 (0.48)	<0.001	LSL < CCF LSL < ADCF
Post-infection changes in socioeconomic factors					
Changes in deprivation index, mean (s.d.)	−0.168 (1.60)	0.143 (2.18)	0.64 (3.85)	<0.001	LSL < ADCF
Any new domestic violence, %	0.7	1.9	4.0	0.018	LSL < ADCF
Currently in full-time employment, %	18.1	26.7	24.0	0.023	LSL < CCF
Current mental well-being					
PHQ-9, median (IQR)	4 (5)	9 (6)	14 (7.5)	<0.001	LSL < CCF <ADCF
GAD-7, median (IQR)	1 (4)	6 (6)	10 (8.5)	<0.001	LSL < CCF <ADCF
ISI, median (IQR)	6 (6)	12 (6)	16 (8)	<0.001	LSL < CCF LSL < ADCF
CFS, bimodal score median (IQR)	3 (6)	8 (4)	9 (3.5)	<0.001	LSL < CCF LSL < ADCF
IES-R, median (IQR)	3 (9)	18 (19)	28 (26.5)	<0.001	LSL < CCF <ADCF
Health-related quality of life					
WHOQOL-BREF (physical), median (IQR)	67.9 (21.5)	53.6 (17.9)	46.4 (17.8)	<0.001	LSL > CCF >ADCF
WHOQOL-BREF (psychological), median (IQR)	54.2 (20.9)	41.7 (20.9)	33.3 (18.8)	<0.001	LSL > CCF >ADCF
WHOQOL-BREF (social), median (IQR)	58.3 (25.0)	50.0 (16.6)	41.7 (16.7)	<0.001	LSL > CCF >ADCF
App based cognitive task performance					
PVT reaction time in ms, median (IQR)	373 (55)	489 (67)	375 (75)	<0.001	LSL < CCF
DSST reaction time of correct trials in ms, median (IQR)	1297 (0.364)	1364 (0.479)	1401 (0.407)	0.0028	LSL > CCF LSL > ADCF
DSST, count of correct trials	62 (18)	59 (21)	58 (19)	0.014	
Two-back task, count of correct trials, median (IQR)	57 (16)	56 (16)	52 (27)	0.026	LSL > ADCF
Alternate finger task, count of correct trials, median (IQR)	39 (35)	33 (29)	33 (26)	0.0028	LSL > CCF LSL > ADCF

LSL, low-symptom-load group; CCF, cognitive complaints–fatigue; ADCF, anxiety–depressive and cognitive complaints–fatigue; IQR, interquartile range; mRNA, messenger ribonucleic acid; PHQ-9, nine-item Patient Health Questionnaire; GAD-7: Generalized Anxiety Disorder-7; ISI: Insomnia Severity Index; CFS, Chalder Fatigue Scale; IES-R, revised version of the Impact of Event Scale; WHOQOL-BREF, World Health Organization Quality of Life Scale; PVT, psychomotor vigilance task; DSST: digit symbol substitution test.

With respect to mental and physical health at the time of the assessment, the LSL group had the least self-reported mental health distress across all measures and the best health-related quality of life (Table 2). The ADCF group scored the worst on most of the mental health and health-related quality of life measures but did not score significantly worse than the CCF group in terms of level of insomnia (Insomnia Severity Index median 6 *v*. 12 *v*. 16 for LSL *v*. CCF *v*. ADCF, respectively; *P* < 0.001; *post hoc* test LSL < CCF, LSL < ADCF) or fatigue (Chalder Fatigue Scale median 3 *v*. 8 *v*. 9 for LSL *v*. CCF *v*. ADCF, respectively; *P* < 0.001; *post hoc* test LSL < CCF, LSL < ADCF). The CCF and ADCF groups had higher current sleep demand than the LSL group (8.0 *v*. 8.4 *v*. 8.5 h for LSL *v*. CCF *v*. ADCF, respectively; *P* < 0.001; *post hoc* test LSL < CCF, LSL < ADCF), although their pre-infection sleep demand was identical. However, the CCF and ADCF groups had shorter sleep duration (median 6.9 *v*. 6.3 *v*. 6.1 h for LSL *v*. CCF *v*. ADCF, respectively; *P* < 0.001; *post hoc* test LSL > CCF, LSL > ADCF), longer sleep latency (median 10 *v*. 15 *v*. 20 min for LSL *v*. CCF *v*. ADCF, respectively; *P* < 0.001; *post hoc* test LSL < CCF, LSL < ADCF, CCF < ADCF) and more midnight arousal (mean 0.8 *v*. 1.2 *v*. 1.6 for LSL *v*. CCF *v*. ADCF, respectively; *P* < 0.001; *post hoc* test LSL < CCF, LSL < ADCF) than the LSL group according to their sleep diaries (Table 2).

The CCF group performed worse than the LSL group in terms of reaction times on the psychomotor vigilance test (mean 373 *v*. 489 *v*. 375 ms for LSL *v*. CCF *v*. ADCF, respectively; *P* = <0.001; *post hoc* test LSL < CCF) and digit symbol substitution test (median 1297 *v*. 1364 *v*. 1401 ms for LSL *v*. CCF *v*. ADCF, respectively; *P* = 0.0028; *post hoc* test LSL < CCF, LSL < ADCF) and on the alternate finger tapping (correct count, median 39 *v*. 33 *v*. 33 for LSL *v*. CCF *v*. ADCF, respectively; *P* = 0.0028; *post hoc* test LSL > CCF, LSL > ADCF), whereas the ADCF group performed worse on the digit symbol substitution test reaction time (see above), 2 back task (correct count, median 57 *v*. 56 *v*. 52 for LSL *v*. CCF *v*. ADCF, respectively; *P* = 0.026; *post hoc* test LSL > ADCF) and alternate finger tapping task (see above) (Table 2).

### Shared but also distinct predictors for CCF and ADCF subgroups

Using multiple logistic regression and controlling for demographic factors, pre-infection physical and mental health status, pre-infection socioeconomic factors, clinical factors related to index acute COVID and psychosocial stressors due to COVID, we found that pre-infection deprivation level and total quarantine duration related to the pandemic were predictive of both CCF (adjusted odds ratio 1.39, 95% CI: [1.10, 1.76], adjusted *P* = 0.025; and adjusted odds ratio 1.04, 95% CI [1.01, 1.06], adjusted *P* = 0.023, respectively) and ADCF (adjusted odds ratio 1.76, 95% CI [1.27, 2.43], adjusted *P* = 0.0054; and adjusted odds ratio 1.05, 95% CI [1.02, 1.08], adjusted *P* = 0.0054, respectively) group status. Number of known medical illnesses pre-infection (adjusted odds ratio 1.63, 95% CI [1.26, 2.11], adjusted *P* = 0.003), moderate-to-severe acute COVID severity (adjusted odds ratio 1.76, 95% CI [1.20, 2.59], adjusted *P* = 0.023) and having had a full-time job before the infection (adjusted odds ratio 2.0, 95% CI [1.32, 3.05], adjusted *P* = 0.01) preferentially predicted CCF status, whereas lack of pre-infection suicidal ideation negatively predicted ADCF status only (adjusted odds ratio 0.18, 95% CI [0.09, 0.34], adjusted *P* < 0.001) (Table 3).

**Table 3:** Multinomial logistic regression: predictors of high-symptom-load phenotypes, with the low-symptom-load group as reference group

Variable	The CCF phenotype (with predominantly CCF cluster symptoms)				The ADCF phenotype (with high numbers of symptoms across symptom clusters)			
	Adjusted odds ratio		95% CI	Adjusted *P*-value	Adjusted odds ratio		95% CI	Adjusted *P*-value
Constant	0.06	0.02	0.26	0.0030	0.20	0.03	1.22	0.32
Demographic factors								
Age in years	1.00	0.98	1.02	0.93	1.01	0.98	1.04	0.67
Female gender	1.45	1.00	2.10	0.15	1.01	0.60	1.71	0.96
Non-Chinese ethnicity	1.26	0.45	3.48	0.82	0.47	0.05	4.49	0.60
Tertiary education	0.95	0.60	1.52	0.93	0.57	0.29	1.09	0.32
Body mass index > 30	1.44	0.73	2.82	0.49	0.55	0.15	1.99	0.56
Pre-infection physical health								
Smoking	1.15	0.57	2.30	0.82	0.73	0.26	2.07	0.60
Number of known medical illnesses	**1.63**	**1.26**	**2.11**	**0.0030**	1.40	0.93	2.11	0.35
Pre-infection mental health								
Known mental illness	1.88	0.95	3.73	0.18	1.36	0.54	3.42	0.60
Absence of suicidal ideation	0.58	0.32	1.05	0.18	**0.18**	**0.09**	**0.34**	**5.6E-06**
Risk of alcoholism	0.43	0.05	3.50	0.64	1.67	0.31	9.10	0.60
Pre-infection socioeconomic factors								
Deprivation index (log-transformed)	**1.39**	**1.10**	**1.76**	**0.025**	**1.76**	**1.27**	**2.43**	**0.0054**
Number of dependent children	1.24	1.01	1.53	0.15	1.22	0.89	1.69	0.45
Absence of domestic violence	0.61	0.28	1.32	0.37	0.44	0.19	1.03	0.29
Number of elderly dependants	1.19	0.91	1.56	0.37	1.33	0.92	1.92	0.36
Full-time work pre-infection	**2.00**	**1.32**	**3.05**	**0.010**	1.51	0.84	2.72	0.42
Clinical factors								
Moderate-to-severe COVID	**1.76**	**1.20**	**2.59**	**0.023**	2.03	1.18	3.51	0.066
Number of mRNA vaccines pre-infection	1.00	0.82	1.22	0.98	0.85	0.65	1.11	0.45
Number of non-mRNA vaccines pre-infection	1.17	0.93	1.48	0.36	0.88	0.64	1.23	0.60
Two or more infections	1.19	0.79	1.77	0.63	1.31	0.74	2.31	0.56
Use of antiviral medication	1.76	0.90	3.47	0.23	0.65	0.18	2.40	0.60
Psychosocial stressors secondary to COVID								
Duration of hospital isolation	0.97	0.87	1.08	0.81	0.95	0.83	1.10	0.60
Duration of quarantine in facilities	1.00	0.97	1.03	0.93	1.02	0.98	1.05	0.56
Duration of quarantine at home	**1.04**	**1.01**	**1.06**	**0.023**	**1.05**	**1.02**	**1.08**	**0.0054**
Number of COVID-related deaths in close social circle	1.17	0.75	1.84	0.67	0.62	0.30	1.30	0.45

CCF, cognitive complaints–fatigue; ADCF, anxiety–depressive and cognitive complaints–fatigue; mRNA, messenger ribonucleic acid.

Bold text indicates adjusted *P*-value <0.05.

## Discussion

To the best of our knowledge, this is the first study to use a pre-registered protocol to replicate the clustering of chronic PCNPS in cohorts infected by different SARS-CoV-2 virus variants. This is also the first study to phenotype patients infected during the Omicron wave with chronic PCNPS by their symptom profiles and to examine their demographical, socioeconomic, health and clinical risk factors.

### Neuropsychiatric symptoms in patients infected with Omicron strains of SARS-CoV-2 more than 1 year ago showed a similar clustering pattern to symptoms in patients infected with pre-Omicron strains

The network analysis results for our Omicron wave cohort replicated the clustering pattern we found in our smaller, pre-Omicron cohort. This supported our primary hypothesis, as both cohorts contained a CCF cluster, a depression–anxiety symptoms cluster and a headache–dizziness cluster, with highly comparable cluster membership. In recent years, failures to replicate results of scientific studies, especially in the fields of psychiatry and psychology, have raised concerns.^11^ Validation in independent cohorts is a gold standard for ensuring generalisability of findings. Understanding the relationships and groupings among symptoms is fundamental to patient stratification and, subsequently, biomarker discovery and interventional studies, as heterogeneity within a study population introduces noise and reduces power. For example, a large intervention trial of probiotics for long COVID symptoms found that these were effective for fatigue and cognitive complaints but not for mood complaints.^12^

### Differential natural clinical and socioeconomic trajectories for different phenotypes

The CCF and ADCF phenotypes had distinct symptom profiles. The presence of the CCF phenotype that had only mild levels of anxiety and depression was particularly intriguing, as persistent fatigue and cognitive complaints are often attributed to the presence of depression and anxiety in the literature;^13^ our results suggest that this is not the case in at least a substantial subset of patients. There were also differences between the CCF and ADCF groups in terms of pre-infection socioeconomic and health status before the infection. These discrepancies increased after infection, with both the CCF and ADCF phenotypes showing increases in physical and mental health problems. The ADCF group particularly suffered most socially, with increased domestic violence and deprivation. However, our analysis limited our ability to infer any causal relationships among post-infection health burden, socioeconomic changes and the presence of chronic PCNPS.

### Pre-existing health vulnerabilities and clinical severity of COVID predict different phenotypes

Our results echo existing evidence that pre-existing mental health, physical health and clinical severity of acute COVID predict the presence of chronic post-COVID symptoms, including but not limited to neuropsychiatric symptoms.^14,15^ However, this is the first study to suggest that different clinical factors predict different chronic PCNPS phenotypes. Predominantly CCF cluster symptoms were predicted by physical factors such as pre-existing physical illnesses and the severity of acute COVID infection. Although post-acute COVID symptoms can occur in patients with mild acute COVID, the clinical severity of acute COVID has been shown to be a predictive factor in some studies.^14,16^ It has been hypothesised that the more severe the initial infection, the more severe the immune dysregulation and/or endothelial dysfunction. Pre-existing physical health problems are also known to increase the risk of more severe COVID.^17^ However, how pre-existing health problems contribute to post-COVID neuropsychiatric problem symptoms after adjusting for acute infection severity is not well understood. Studies in different contexts have suggested that people with multiple morbidities tend to report higher levels of cognitive complaints and fatigue^18^ and even functional neurological symptoms.^19^ Therefore, it is difficult to ascertain the role of COVID in patients complaining of prolonged fatigue if they also have multiple medical comorbidities. Notably, the severity of acute COVID and the number of pre-infection morbidities predicted only the CCF phenotype, not the ADCF phenotype. On the contrary, the ADCF phenotype was predicted by pre-infection presence of suicidal ideation, a proxy marker of poor mental health. This distinction in risk factors between the two phenotypes suggests that different chronic PCNPS phenotypes might have different underlying mechanisms. A recent longitudinal study found that pre-existing psychiatric problems, but not acute infection severity, were predictive of long-term neuropsychiatric outcomes in a group of patients with a high prevalence of a mixture of post-COVID persistent depression, anxiety, fatigue and cognitive impairment; this echoes our findings regarding predictors of the ADCF phenotype.^20^ In other words, some inconsistency in the literature concerning risk factors for post-COVID symptoms may be partly explained by a lack of recognition of distinct phenotypes among patients.

### Health gap

Deprivation is known to predict negative health outcomes in a wide range of contexts, and the public health crisis of the COVID pandemic has been no exception: more deprived populations had higher mortality rates,^21^ poorer access to healthcare resources,^22^ worse psychosocial stress during the pandemic,^23^ and so on. Hastie et al^24^ found that those who had deterioration of health post-COVID in a large UK cohort were more deprived, after adjusting for potential confounders. Our previous study showed that the level of deprivation before infection predicted higher loading of chronic PCNPS in a separate, pre-Omicron cohort,^4^ and pre-infection deprivation also predicted both the CCF and ADCF phenotypes in our Omicron wave cohort, supporting our hypothesis. This replicable result suggests that social mechanisms of chronic PCNPS should not be overlooked in the quest to understand this phenomenon.

### The vaccination question

Contrary to our hypothesis, we did not find vaccination to be protective against chronic PCNPS. Studies generally agree on the positive protective effect of vaccination against post-COVID symptoms,^25–27^ with a large-scale database study by Lundberg-Morris et al^28^ demonstrating that vaccination was least effective in preventing post-COVID symptoms in patients infected with the Omicron variant. However, most of these studies used a shorter time frame of 4 weeks to 6 months to define post-COVID symptoms. Some studies of longer-term outcomes (>1 year) showed negative results.^29^ We found a protective effect of pre-infection COVID vaccination on chronic PCNPS in our study of the pre-Omicron cohort, but not in the current Omicron wave cohort. Whether this discrepancy was related to the virulence of the different strains, the reduced effectiveness of first-generation COVID vaccine against later strains, the chronicity of the symptoms or a combination of these factors cannot be ascertained at this point.

### The lasting effect of prolonged quarantine

Previous studies on past pandemics and/or epidemics have shown a relationship between longer quarantine periods and poor mental health outcomes,^30^ and multiple studies found that quarantine was associated with an increase in mental health distress during the COVID pandemic.^31,32^ Quarantine affects mental health by increasing social isolation and disruption of daily routine. In Hong Kong, where living space is limited, home quarantine can be particularly stressful and can increase conflict within the household. However, although we found a statistically significant association between the duration of home quarantine and the presence of chronic PCNPS, the odds ratio was very small (1.04–1.05), and the real-world significance of this result is doubtful. It is possible that the psychosocial effect of prolonged home quarantine may have been diluted over time.

### The enigma of chronic PCNPS and its clinical implications

The knowledge gap with respect to chronic PCNPS remains enormous, but the current study represent a step forward; by uncovering different phenotypes of chronic PCNPS using scientifically sound methodology, it has enabled us to pursue a phenotype-driven approach to understanding divergent patient characteristics and risk factors. We believe this phenotype-driven approach will be a promising strategy for further biomarker discovery studies and treatment trials based on specific hypotheses for the mechanisms of syndrome(s), for example, the immune dysregulation hypothesis. We also believe that such an approach could improve the signal-to-noise ratio in the literature and potentially provide information about phenotype-specific aetiologies and treatment options for personalised medicine. From a clinical perspective, it is important for clinicians to recognise the existence of distinct phenotypes among patients complaining of chronic PCNPS, so that they can distinguish patients with higher psychosocial needs (those with the ADCF phenotype) from those with the neurocognitive–fatigue-predominant CCF phenotype and prioritise the appropriate care accordingly. The insights we have obtained here with respect to PCNPS are potentially applicable to other post-infection neuropsychiatric syndromes, especially in the context of emerging infectious diseases or fast-evolving epidemics when we need to navigate through noisy data and respond with swift public health measures.

A phenotype-driven approach will also be helpful in further research to investigate the pathophysiology of chronic PCNPS and the effectiveness of interventions. In addition to biological factors, psychosocial mechanisms and their roles in chronic PCNPS require further research.

### Strengths and limitations

The major strength of this work was that it is a registered study with pre-specified hypotheses and assessment methods. Furthermore, it was in part a replication of another study involving a different cohort, which improved the generalisability of the results. Statistically, we used multiple testing corrections in our multiple regression analysis results, an approach that can reduce false positive rates. The study’s major limitation was the convenience sampling method, which can introduce sampling bias. We also relied on self-reported infection status to determine whether participants belonged to the Omicron wave cohort; this was the key eligibility criterion, and we could not rule out false reporting. We also did not have access to the medical records of the participants. Our assessment relied mostly on self-report measures. In addition, the app-based cognitive test covered key measures focusing on the ‘brain fog’ phenomenon but did not cover all cognitive domains. We also did not have normative data on the performance of the tasks in the general population for comparison. Finally, this study used a cross-sectional design, and the data on participants’ pre-infection and infection status were subject to recall bias. This design also limited our ability to infer causal relationships between predictors and target outcomes.

## Supporting information

Chau et al. supplementary material Chau et al. supplementary material

## Data Availability

The data that support the findings of this study will be openly available after the paper is published.

## Supplementary material

The supplementary material is available online at https://doi.org/10.1192/bjo.2025.10078
